# Dynamic Synchronization and Resonance as the Origin of 1/f Fluctuations—Amplitude Modulation Across Music and Nature

**DOI:** 10.3390/e28010038

**Published:** 2025-12-27

**Authors:** Akika Nakamichi, Izumi Uesaka, Masahiro Morikawa

**Affiliations:** 1 General Education, Kyoto Sangyo University, Motoyama, Kamigamo, Kita-ku, Kyoto 603-8555, Japan; nakamichi@cc.kyoto-su.ac.jp; 2 SFIDA X Osaka Office, Sonezaki Shinchi, Kita-ku, Osaka 530-0002, Japan; mafuizu@outlook.jp; 3 RIKEN, Wako, Saitama 351-0198, Japan

**Keywords:** 1/f fluctuation, music, synchronization, resonance, amplitude modulation

## Abstract

In natural systems, astrophysics, biological physics, and social physics, 1/f fluctuations are observed across a wide range of systems. Focusing on the case of music, we propose and verify a physical mechanism for generating these fluctuations. This mechanism is based on amplitude modulation (AM) and demodulation (DM), where the 1/f spectral law appears not in the raw waveform but in its demodulated amplitude envelope. Two distinct yet complementary processes generate the required AM: (i) stochastic synchronization among oscillators, modeled via an extended Kuramoto framework that captures perpetual synchronization–desynchronization cycles, and (ii) frequency-selective resonance, modeled by spectral accumulation of eigenmodes in acoustic or structural environments. Numerical simulations demonstrate that both mechanisms, acting alone or in combination, robustly generate 1/f spectra spanning several digits when demodulation is applied and that the classical Kuramoto critical point is not essential for its emergence. While this analysis focuses on 1/f fluctuations in musical performance and acoustics, we also note that 1/f fluctuations inherent in musical scores may be similarly described by the AM/DM mechanism.

## 1. Introduction

Also known as pink noise, 1/f fluctuation is a ubiquitous phenomenon that is widely observed and is characterized by the low-frequency power law $S\left(\omega \right)\propto \omega^{-\beta }$ in the power spectral density (PSD), where ω is frequency and β=0.5∼1.5. These 1/f fluctuations appear everywhere in nature, such as in semiconductors, thin metals, bio-membranes, crystal oscillators, heartbeat rates, magnetoencephalography (MEG), and electroencephalograms (EEG) (brain), etc. [1]. Furthermore, artificial systems, such as music sound data, often exhibit typical 1/f fluctuations. This ubiquity strongly suggests the existence of a common underlying physical mechanism, yet its precise nature remains a subject of debate.

Since the first discovery of 1/f fluctuations in 1925 in the voltage fluctuations in a vacuum tube [2], diverse theoretical explanations were proposed, including self-organized criticality, fractional Brownian motion, percolation models, and aggregation–relaxation processes [1]. While these frameworks successfully describe certain systems, they lack universality: Each model tends to be domain specific and often requires fine-tuning to reproduce empirical scaling exponents. A single, simple, and scalable physical principle that can generate 1/f spectra across such varied contexts has yet to be established. We argue that such ubiquitous phenomena must stem from simple and universal physical principles rather than complex, system-specific ones.

In our previous publications [3,4], we proposed that the origin of 1/f fluctuations is the wave beat, or amplitude modulation (AM). In particular, if many waves with frequencies closer to each other can exhibit signals with arbitrarily low frequencies. Then, we attempted to verify and extend this proposal by applying it to earthquakes [5], solar flares [6], electric currents [7], and other phenomena. These systems were too complex to directly verify the simple mechanism.

In this paper, we focus on music exhibiting typical 1/f fluctuations. There have been a tremendous number of works on music 1/f fluctuations after the initial work [8,9]. We examine the extent to which the above unified framework of AM applies to music. Music is generally composed of three layers: (A) Structure: Musical score is the composer’s creative output and describes the basic structure of the music. (B) Physiology: Players’ performance emits sound based on the score and exhibits their interpretation of the music. (C) Physics: The emitted sound resonates and propagates toward the listeners in the hall. We analyze the final output signal after all these layers in relation to the layers (B) and (C). Although some research reports the presence of 1/f fluctuations in the first layer (A), this layer is closely related to human brain activity and falls beyond our present scope [10]. We will extend the discussion on how to analyze this layer at the end of this paper.

The key physical requirement for the AM of 1/f fluctuations is frequency accumulation, i.e., the presence of many oscillatory components with closely spaced frequencies, leading to slow envelope modulations via wave beating. We identify two distinct yet complementary physical processes that naturally generate such frequency accumulation:

1. Synchronization: Stochastic synchronization among interacting oscillators leads to recurrent cycles of phase alignment and dispersion. We model this process using an extended stochastic Kuramoto framework that produces persistent low-frequency envelope variations without requiring fine-tuning of the classical synchronization threshold.
2. Resonance: Resonance-driven spectral shaping, in which environmental or structural eigenmodes selectively amplify certain frequencies, creates envelope modulations even in the absence of direct coupling among oscillators.

Our approach offers three major departures from previous works: First, it unifies the synchronization-based and resonance-based origins of 1/f noise within a single dynamical framework. Second, it shows that criticality is not a prerequisite: 1/f spectra arise over broad parameter ranges. And third, it demonstrates the cross-domain applicability of the AM/DM mechanism through numerical simulations and analyses of real-world data from orchestral and solo musical performances, seismic records, and astrophysical time series.

The remainder of this paper is organized as follows: Section 2 summarizes empirical observations and unresolved puzzles regarding 1/f fluctuations in musical signals. These turn out to be good starting points for finding a simple physics for the origin of 1/f fluctuations. Section 3 introduces the stochastic Kuramoto model as a description of synchronization-induced AM and presents numerical results. Section 4 develops a resonance-based AM model and validates it against measured acoustic environments. Section 5 integrates the two mechanisms into a unified framework and explores their interplay in generating 1/f fluctuations. Section 6 presents possible approaches to the music layer (A) and part of (B) that build upon the successful application demonstrated in the previous sections. Finally, Section 7 discusses implications, limitations, and potential extensions of the present theory.

## 2. A Simple Origin of 1/f Fluctuations—Amplitude Modulation

**Figure 1:**
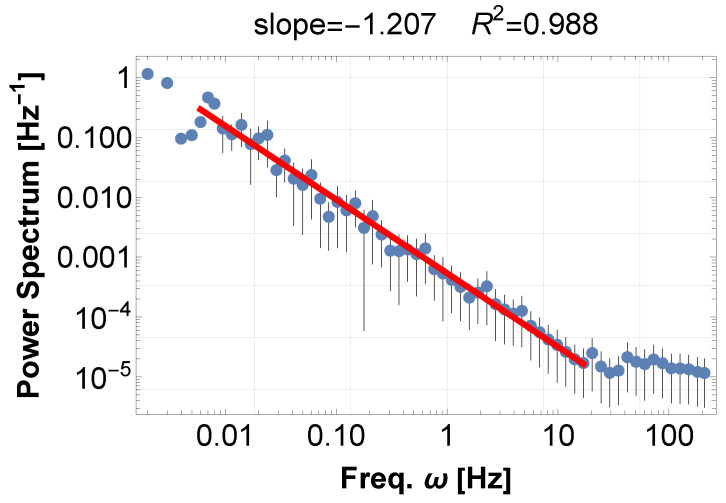
PSD of the sound data from Tchaikovsky’s Symphonie no 6 en si mineur, “pathétique” for Strings [11]. The total number of data points is 44,251,704, which is divided into 442,517 sections, and in each of these, the sum of each data point squared corresponds to a sampling rate of 441 per second.The graph displays a power law with an index of −1.21 for about four digits. The total duration is 1003.44 s. The blue dots represent the FFT points equally distributed on a log scale, and the red line represents the best-fit power law to the data. The same applies to the other figures in this paper. The detail is explained in the text. We consistently avoid using window functions for the reasons explained in the text. However, for reference, analyzing the results yields the following exponents for no window, Han window, and Hamming window, respectively, with the exponent fluctuating by a maximum of 1%: $-1.207\left(R^{2}=0.988\right),\;-1.198\left(R^{2}=0.984\right)$, and $-1.197(R^{2}=0.983)$.

**Figure 2:**
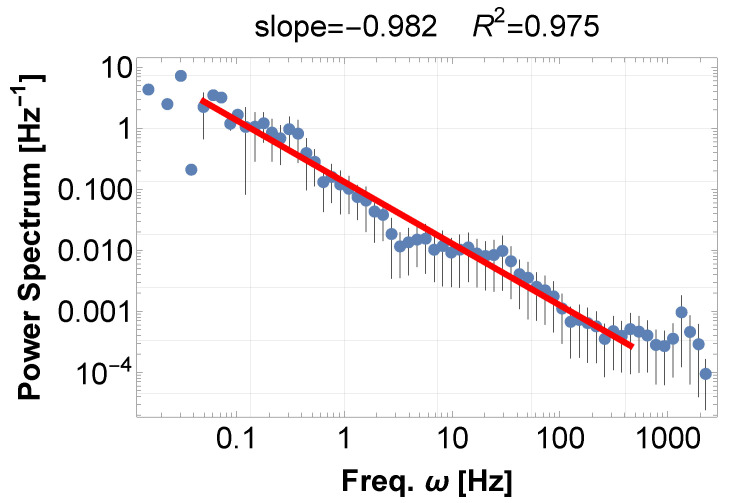
PSD of the sound data recorded at the Chamber Music Concert, OAC, 12 July 2023, by Kalliopi Petrou (soprano), Stefano Menegus (Piano) [12]. The power-law fit (red) displays a power index of −0.98 to more than four digits. We consistently avoid using window functions for the reasons stated in the main text, but for reference, analyzing the results yields the following exponents for no window, Han window, and Hamming window, respectively, with the exponent fluctuating by a maximum of 9%: $-0.982\left(R^{2}=0.975\right),-1.071\left(R^{2}=0.965\right)$, and $-1.076(R^{2}=0.963)$.

For a typical example of musical sound, we show (1) an orchestra music concert [11], and (2) soprano and chamber music concert [12]. Their PSDs exhibit typical 1/f fluctuations, with indices of −1.21 in Figure 1 and −0.98 in Figure 2, to more than four digits in both cases. Later, these examples turn out to be typical realizations of frequency accumulation—synchronization and resonance, respectively.

Here we briefly describe our PSD analysis. We do not apply window functions before PSD analysis. Window functions are primarily designed to suppress spectral leakage arising from discontinuities at the boundaries of finite-length signals. While effective for identifying narrowband spectral features, they can distort broadband scale-free spectra by convolving the true power-law spectrum with the window’s spectral response, particularly affecting the low-frequency regime. For the purpose of estimating global power-law scaling, such as 1/f noise, the use of window functions may introduce systematic bias in the inferred exponent, and therefore, is not necessarily advantageous. As a matter of fact, we have checked Hann and Hamming windows and obtained almost the same power index—within several percent.

To reduce statistical fluctuations while preserving scale-free behavior, the raw PSD was averaged over logarithmically spaced frequency bins. The error bars represent the variance within the bin. Power-law scaling was assessed by performing sequential linear regressions on the log–log binned PSD, disregarding several of the lowest-frequency points, starting from the lowest frequency and progressively including higher-frequency bins. For each cumulative fitting range, the power-law exponent and the coefficient of determination $R^{2}$ were evaluated, and the range yielding the maximum $R^{2}$ (subject to $R^{2}\geq 0.5$ and a stable exponent) was selected. The red line was thus selected as the best fit within this range. The corresponding exponent was adopted as the final estimate of the spectral scaling. The remaining PSD graphs in this paper were obtained using the same method.

The uniformity of the time-series data may be a concern. In general, the data is not uniform, and the 1/f fluctuation property is not steady; thus, the PSD here describes the average properties of the system. However, this non-uniformity itself is a powerful tool for observing the system much further. See Section 7 and the figure therein.

We now examine the fundamental features of 1/f fluctuations in music. There are at least three unique properties of 1/f fluctuations in the music.

1. Low-frequency signal continues without limit: The power −1 in PSD continues without bound. This implies a divergence in total power, as each octave contributes equally to the energy. Furthermore, if stationary, the system appears to possess an infinitely long memory, according to the Wiener–Khinchin theorem, which relates the PSD to the time correlation function. In particular, the orchestral music exhibits 1/f fluctuations throughout its entire performance, for example, for more than 30 min in Figure 1. This is contrary to the case in layer (A), where the domain of the power −1 in the PSD is one second to a few minutes (0.01 to 1 Hz) [10].
2. Arbitrary low-frequency signal arises from a tiny system. In the ordinary argument, the system size determines the limiting frequency by a general-order estimate. From the system size *l* and the typical wave speed *v*, the maximum correlation time scale is of the order l/v. However, the 1/f fluctuation in music violates this general rule. The 100 m music hall and the sound speed only yield a characteristic timescale of 0.3 s or several Hz. On the contrary, the fluctuation of music 1/f continues for an hour or more. Therefore, we speculate that 1/f fluctuation in music is not an intrinsic property but something secondary among many waves, such as interference between them.
3. In the music, 1/f fluctuation appears in the PSD for the data squared or zero-crossing time series; the original data never shows 1/f fluctuations. This fact has been widely appreciated since the pioneering work [8], as if it were self-evident. However, this is strange, as the original layer (A) directory exhibits 1/f fluctuations without any manipulation. Sometimes extra 1/f fluctuations may be generated in the later layers (B) and (C). The same property, the necessity of square operation, appears in economic data and some astrophysical 1/f fluctuations, while many others exhibit direct 1/f fluctuations. What is the difference?

Let us first examine Property 3. We perform other operations on the original sound signal, such as arbitrary-power operations, rectification, and thresholding on the original orchestra music data of Figure 1. Then, we found the following results of arbitrary power operation, as shown in Table 1.

These results naturally suggest that the square operation in Property 3 is a kind of demodulation (DM) operation of the encoded 1/f fluctuations. Rapidly alternating positive and negative values in the original sound data cancel each other out, leaving no information about the encoding.

Suppose that two signals with near frequencies (0<λ≪ω) are superposed,

(1) $$g=\sin\left(\left(\lambda +\omega \right)t\right)+\sin\left(\left(-\lambda +\omega \right)t\right)=\underset{A(t)}{\underset{︸}{2\cos(\lambda t)}}\sin\left(\omega t\right),$$

and the low-frequency signal A(t) is on the sine transportation wave: A(t)sin(tω). The *n*-th power of this becomes (n=1,2,…)

(2) $$\begin{array}{cc} g & =A(t)\sin(t\omega )\\ g^{2} & =\frac{1}{2}A{\left(t\right)}^{2}\left(1-\cos(2t\omega )\right)\\ g^{3} & =\frac{1}{4}A{\left(t\right)}^{3}\left(3\sin(t\omega )-\sin(3t\omega )\right)\\ g^{4} & =\frac{1}{8}A{\left(t\right)}^{4}\left(3-4\cos(2t\omega )+\cos(4t\omega )\right)\\ g^{5} & =\frac{1}{16}A{\left(t\right)}^{5}\left(10\sin(\omega t)-5\sin(3t\omega )+\sin(5t\omega )\right)\\ \; & \ldots \end{array}.$$

The isolated term of $A{\left(t\right)}^{n}$ exists in the even-*n*-th power, while $A{\left(t\right)}^{n}$ mixes with the transportation wave in the odd-*n*-th power. The binomial expansion $\sin{\left(x\right)}^{n}={\left(\left(e^{ix}-e^{-ix}\right)/\left(2i\right)\right)}^{n}$ generally proves this property. Furthermore, this even/odd-power property is also true for signals that include overtones in their timbre. The similar expansion ${\left(\sin\left(x\right)+\sin\left(2x\right)\right)}^{n}$ proves this fact. Thus, we can naturally infer that the origin of 1/f fluctuation is the wave beat or the amplitude modulation (AM). We further hypothesize that 1/f fluctuations appear after any demodulation operation (DM).

We verified other DM operations, such as absolute value and thresholding based on the mean absolute value of the data, as shown in Table 2. All possible DMs applied to the original sound data appear to extract 1/f fluctuations successfully.

If AM-DM were the origin of 1/f fluctuations, Properties 1 and 2 are naturally obtained as a result of the wave beat associated with AM. Although the original data, as shown in Equation (1), exhibits only two peaks in the PSD at the original frequencies, the square of the data exhibits an extra beat peak at an arbitrary low frequency at 2λ.

The beat frequency λ can be arbitrarily small. A typical example is the oldest electric instrument, the theremin, which has two nearly identical high-frequency generators: One is of fixed frequency, and the other is slightly controlled by adjusting the capacitance between the player’s hand and the antenna. The wave beat between them yields an audible sound.

As pointed out in Property 3 above, music data often exhibits pink noise when squared, and even when analyzing the PSD of the zero-crossing time series derived from the original data’s zero crossings, it still appears to show pink noise. The former can be explained by frequency accumulation causing beat effects, AM. On the other hand, the latter zero-crossing time series is equivalent to frequency modulation (FM).

Based on Equation (1), let us consider FM as follows. The data *g* slowly undulates due to the AM of A(t). Near the peaks of this undulation, the zero crossings occur at roughly constant intervals. However, at troughs of this undulation, A(t) slowly changes its signature, causing the zero crossing intervals nonuniform in that vicinity. In other words, the AM created by the linear superposition of multiple components is strongly coupled to the phase. Thus, the zero-crossing mechanism can be considered as an operation that extracts the nodes (envelope sign reversal) of AM. Consequently, AM and FM are consistent with each other generating 1/f fluctuations.

However, a simple beat is not sufficient to yield a power-law PSD. We further need a systematic frequency accumulation for 1/f fluctuations.

The most typical accumulation of frequencies would be the exponential form: $\omega =e^{-\kappa \xi }$, where κ is a constant and ω is the frequency [4]. The positive variable ξ is uniformly random within some range with a constant probability *p*. Then we immediately have the frequency distribution function inversely proportional to the frequency ω:

(3) $$P\left(\omega \right)=p\left|\frac{d\xi }{d\omega }\right|=\frac{p}{\kappa }\frac{1}{\omega },$$

and the frequency difference (Δω) distribution function Q(Δω) becomes

(4) $$\begin{array}{cc} Q(\Delta \omega ) & =\int_{\omega_{1}}^{\omega_{2}}d\omega P\left(\omega +\Delta \omega \right)P\left(\omega \right)\\ \; & =\frac{p^{2}}{\lambda^{2}\Delta \omega }\ln\left[\frac{\omega_{2}\left(\omega_{1}+\Delta \omega \right)}{\omega_{1}\left(\omega_{2}+\Delta \omega \right)}\right], \end{array}$$

which is inversely proportional to Δω, if we set the lower limit $\omega_{1}$ of the integration sufficiently small. Further, in the case of power law frequency accumulation $\omega =t^{-\alpha }$, the frequency difference distribution function Q(Δω) becomes $Q\left(\Delta \omega \right)\propto \Delta \omega^{-1-(2/\alpha )}$: the fiducial power is still −1 [4]. Based on this principle, we superpose many (N) sine waves with frequencies approaching each other exponentially,

(5) $$\phi \left(t\right)=\sum_{i=1}^{N}\sin\left(2\pi \omega \left(1+ce^{-\xi_{i}}\right)t\right),$$

where $\xi_{i}$ is a uniform positive random variable, and ω,c are constants. Numerical demonstration shows that the PSD of this wave superposition itself ϕ(t) does not display 1/f fluctuations, while $\phi {\left(t\right)}^{2}$ does. Similar frequency accumulation is also expected for the power-law approach [4].

## 3. Synchronization Mechanism: Orchestral Unison and the Stochastic Kuramoto Model

To model how 1/f fluctuations emerge from the collective behavior of multiple musical performers in unison, we employ a stochastic version of the Kuramoto model (SKM). This model captures the essential dynamics of weakly coupled oscillators that tend to synchronize their phases despite individual differences in natural frequencies and noise perturbations.

### 3.1. The Kuramoto Framework

The classical Kuramoto model [13] describes the evolution of the phase $\theta_{i}\left(t\right)$ of *N* coupled oscillators as:

(6) $$\frac{d\theta_{i}\left(t\right)}{dt}=\omega_{i}+\frac{K}{N}\sum_{j=1}^{N}\sin\left(\theta_{j}\left(t\right)-\theta_{i}\left(t\right)\right),$$

where $\omega_{i}$ is the natural frequency of the *i*-th oscillator, and *K* is the global coupling strength. In a musical analogy, each $\theta_{i}$ represents the phase of a performer’s sound production, and the coupling term represents mutual auditory adjustment among performers.

The collective mode in the Kuramoto model is the mean of the phases:

(7) $$re^{i\psi }=\frac{1}{N}\sum_{i=1}^{N}e^{i\theta_{i}}.$$

For the large *N* limit, the Kuramoto model has a critical point $K_{c}$. The system does not synchronize at all (r=0) for $K<K_{c}$, and gradually, *r* grows for *K* increases beyond $K_{c}$ [13]. Thus, synchronization or desynchronization is fixed by the parameters. In this stationary case, no large fluctuations or 1/f fluctuations are observed.

Human music performance is not at all stationary either. Actual musicians do not achieve synchronization by approaching a target pitch from one side and stopping there. When performing a piece, the performer resets the pitch from scratch each time the melody pitch changes. Musical performance is thus structured as a continuous cycle of synchronization and desynchronization.

To account for natural variation and timing instability among human performers, we introduce random noise [14]:

(8) $$\frac{d\theta_{i}\left(t\right)}{dt}=\omega_{i}+\frac{K}{N}\sum_{j=1}^{N}\sin\left(\theta_{j}\left(t\right)-\theta_{i}\left(t\right)\right)+\xi_{i}\left(t\right),$$

where $\xi_{i}\left(t\right)$ symbolically represents this randomness.

However, this is not the ordinary Gaussian white noise. We introduce intermittent partial randomization events: At random times, we add a uniformly distributed random value from the interval [−1,1] to the phase $\theta_{i}\left(t\right)$ of each oscillator to determine its new phase. If the adding phase were determined within the range [−π,π), it would become completely random, effectively resetting the initial conditions each time. Therefore, we are applying a moderate level of randomization. Ideally, the range should also be analyzed as a variable parameter, but the paper lacks the scope for this; it will be discussed in a future comprehensive paper. This random resetting mimics an abrupt loss of synchrony followed by gradual re-synchronization via coupling. In an ensemble, each performer perpetually transitions from one note to the next. In the simplest model, this is captured by stochastic phase reassignment, representing the onset of the next note without explicit rhythmic structure. The coupling term then realigns performers over time, producing cycles of partial synchronization and resynchronization.

Such resetting-type noise has been studied in non-equilibrium statistical physics and captures intermittent desynchronization phenomena more directly than continuous additive noise [15]. Sometimes, this resetting-type noise itself exhibits non-Gaussian properties. However, such an artificial effect is absent in our case, as the non-trivial PSD arises only after demodulation.

The above is not the sole extension of the Kuramoto model. Since we want to examine general properties of synchronization and 1/f fluctuations, we also consider another form of extension, including the inertia term [16,17]:

(9) $$\frac{d^{2}\theta_{i}\left(t\right)}{dt^{2}}=-\omega_{i}^{2}\theta_{i}\left(t\right)+\frac{K}{N}\sum_{j=1}^{N}\sin\left(\theta_{j}\left(t\right)-\theta_{i}\left(t\right)\right)+\xi_{i}\left(t\right).$$

Actual musicians do not achieve synchronization by approaching the target pitch from one side and stopping there. Instead, they synchronize by repeatedly oscillating—approaching, overshooting, and returning. It is often the case that musical performers intentionally oscillate around the target pitch: This is vibrato. This inertial Kuramoto equation models such a realistic performance.

### 3.2. From Phase to Sound Signal

To construct a musical waveform from this model, we interpret each oscillator as emitting a sine wave whose instantaneous frequency is derived from its phase. Therefore, we use the imaginary part of the above order parameter Equation (7) as the indicator:

(10) $$x\left(t\right)=\frac{1}{N}\sum_{i=1}^{N}\sin\left(\theta_{i}\left(t\right)\right).$$

The aggregated signal x(t) reflects the combined audio signal of the orchestra. Its envelope shows amplitude modulation arising from partial synchronization and beat phenomena among the oscillators.

### 3.3. Numerical Simulation

**Figure 3:**
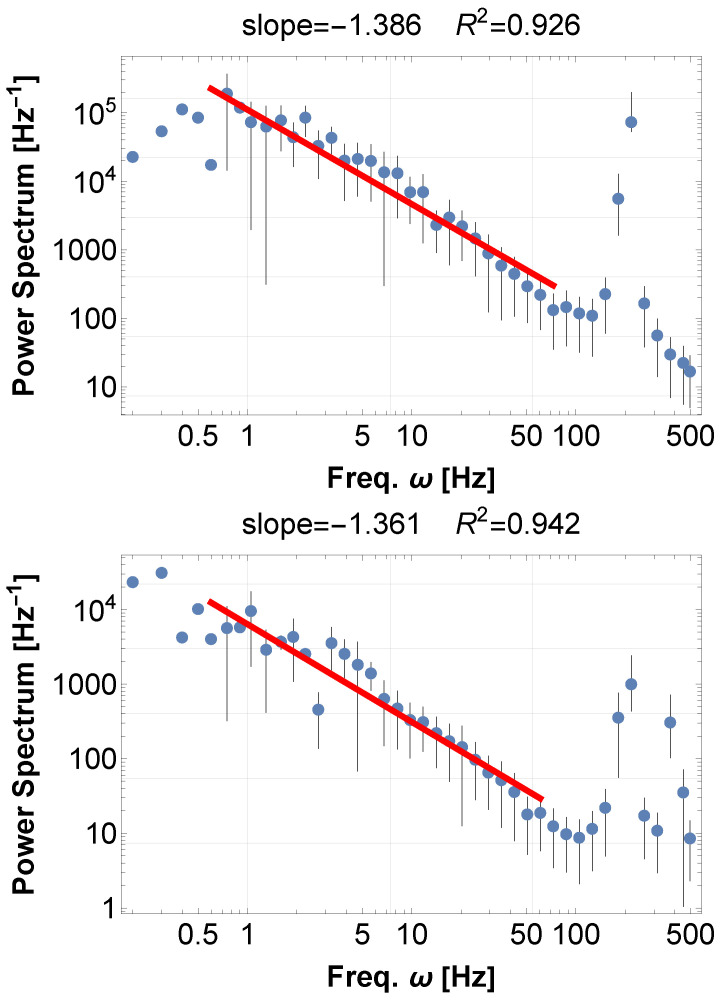
(**Above**) PSD of time series $x{\left(t\right)}^{2}$ calculated from the Kuramoto 1st order equation Equation (8). The blue dots represent the FFT points equally distributed on a log scale, and the red line represents the best-fit power law to the data. The same applies to the other figures in this paper. Parameters are arbitrarily chosen: N=20,K=20, the natural frequencies $\omega_{i}$ are random uniformly distributed around 100 Hz within 1% range, and the time duration is 10. The random force effect, consistently in this paper, is realized by the random shift of the variables $\theta_{i}$ of an amount within the range [−1,1] at a random timing within the interval [0,0.05]. (**Below**) PSD of time series $x{\left(t\right)}^{2}$ calculated from the Kuramoto second-order equation Equation (9), with the same parameters as above. In both cases, 1/f fluctuations naturally arise without particular fine-tuning. Further details will be discussed in Section 5.

We first perform simulations of SKM for some values of N,K, where natural frequencies are uniformly distributed around 100 Hz within a 1% range. After generating the waveform x(t), we compute the power spectral density (PSD) for both the raw and squared signals. There are several methods for simulating the random force effect, but the results for the PSD do not change significantly. The details are in the captions of Figure 3.

While the raw signals only show the clustering frequencies of each element by synchronization, the squared signal reveals a robust 1/f-type power law over several decades, as shown in Figure 3. Interestingly, this property is common to both models, Equations (8) and (9), displaying the generality of the mechanism of 1/f fluctuations from synchronization. Further comparison is described in Section 5.

It is also important to point out the following additional properties of the simulation.

1. The partial sum over several $\sin(\theta_{i}\left(t\right))$ in Equation (10) still shows 1/f fluctuations if squared.
2. Furthermore, a single variable $\sin(\theta_{i}\left(t\right))$ still shows 1/f fluctuations if self-superposed with the delayed data $\sin(\theta_{i}\left(t-\tau \right))$ and squared. This provides a notable contrast, as only a single variable $\sin(\theta_{i}\left(t\right))$ itself does not show 1/f fluctuations, even after being squared.
3. Even a bare superposition of the variables $\theta_{i}\left(t\right)$ shows 1/f fluctuations if squared.

All of the above facts indicate that the 1/f fluctuation is quite robust, based on its interference with other variables or with its history.

**Figure 4:**
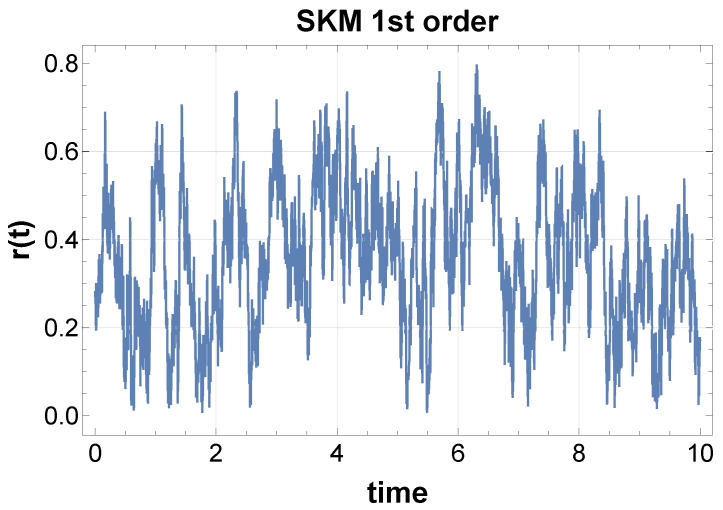
Time evolution of the order parameter r(t) in Equation (7) is strongly fluctuating. Parameters are the same as Figure 3 above.

We want to emphasize that the order parameter Equation (7) is strongly fluctuating, as shown in Figure 4, while *N* is finite; the low-frequency fluctuation is modulated in the order parameter. Furthermore, the system repeats synchronization and desynchronization as shown in Figure 4 in which the synchronization parameter *r* in Equation (7) violently fluctuates.

### 3.4. Interpretation

This simulation shows that when the input frequencies are narrow band, the collective dynamics of perpetual synchronization and desynchronization generate low-frequency modulations. These modulations are then demodulated via squaring, yielding 1/f fluctuations in the amplitude envelope.

Such a mechanism plausibly underlies the 1/f structure in orchestral recordings, where numerous performers with slightly different tempi attempt to maintain unison. The orchestra retakes notes at each pitch transition of the musical melody. In this case, the orchestra synchronizes again at that timing in unison. In this way, music is a series of new synchronizations over and over again.

## 4. Resonance Mechanism: Solo Performance and Acoustic Environments

In contrast to orchestral unison, where amplitude modulation arises from synchronization among performers, solo performances often exhibit amplitude modulation due to *resonance* with the surrounding acoustic environment. This section explores how such resonance-induced amplitude shaping contributes to the emergence of 1/f fluctuations when combined with demodulation.

### 4.1. Resonant Amplification in Physical Systems

Acoustic resonance occurs when a sound wave’s frequency matches the natural frequency of a cavity or structure, resulting in significant amplitude amplification. The frequency response of such systems often follows a Lorentzian profile:

(11) $$H\left(f\right)=\frac{1}{{\left(f-f_{0}\right)}^{2}+{\left(\gamma /2\right)}^{2}}\;.$$

Here, $f_{0}$ is the resonant frequency, and γ characterizes the bandwidth or damping factor. In concert halls or vocal tracts, multiple such modes overlap to create a composite filtering effect.

### 4.2. Solo Sound and Resonance Characteristics Due to Room Reverberation

A solo instrument or voice, when played in a hall, is effectively filtered by the resonance characteristics due to room reverberation (RR). The resulting waveform is amplitude modulated by resonant peaks in RR.

We first calculate the eigenmodes of a typical concert hall, Großer Musikvereinssaal (Goldener Saal) in Vienna, Republic of Austria [18]. This hall is almost cuboid (rectangular prism): depth $L_{1}=48.8$ m, width $L_{1}=19.2$ m, and height $L_{3}=17.75$ m. Thus, the following simple eigenfrequencies are associated with the Helmholtz acoustic equation:

(12) $$f=\frac{v_{s}}{2}\sqrt{{\left(\frac{n_{1}}{L_{1}}\right)}^{2}+{\left(\frac{n_{2}}{L_{2}}\right)}^{2}+{\left(\frac{n_{3}}{L_{3}}\right)}^{2}}.$$

Here, $v_{s}=343$ m/s is the sound speed and $n_{1},n_{2},n_{3}$ run 1,2,…,N. Then, superposing the sine wave of these frequencies,

(13) $$\phi \left(t\right)=\sum_{n_{1},n_{2},n_{3}=0}^{N}\sin\left(2\pi ft\right),$$

and further adding the reflection waves with time delay from each direction, we have the natural acoustic signal in this hall as

(14) $$\phi_{a}\left(t\right)=\sum_{m=1}^{3}\sum_{k=1}^{M}{\left(1+k\right)}^{-\alpha }\phi \left(t-\frac{L_{m}}{v_{s}}k\right),$$

where ${\left(1+n\right)}^{-\alpha }$ with α=0.1 is assumed to be the power reduction rate for each reflection. These settings are a simple trial for our idea of amplitude modulation, although there are many examples of elaboration and research in the field of room acoustics [19,20].

### 4.3. Numerical Illustration

**Figure 5:**
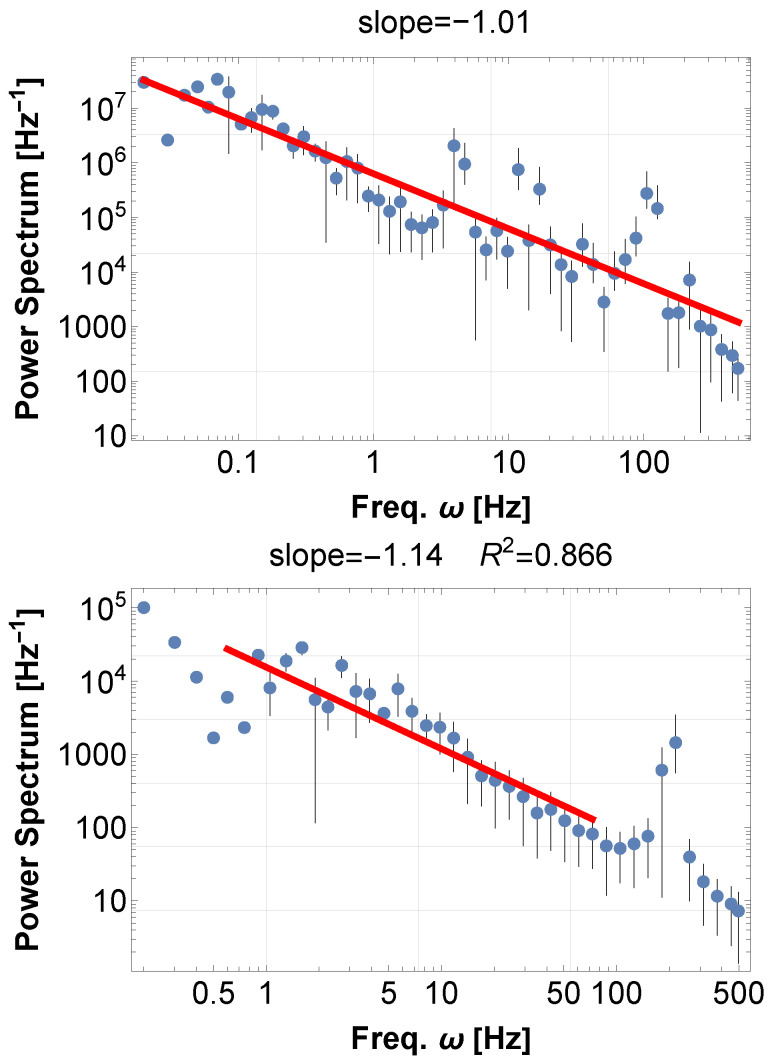
(**Above**) PSD of the Hall resonance. The typical case of Großer Musikvereinssaal (Goldener Saal) in Vienna, Republic of Austria. The resonating sound data is obtained by calculating the eigenfrequencies of the almost cuboid (rectangular prism) Hall Equation (12), adding sine waves with the first 500 modes (13), and further adding the acoustic reverberation of the hall (14).The maximum $R^{2}$ method, as employed in other graphs, was not effective in the present case, probably due to the several wide-band peaks. Therefore, we fit the whole range of frequency to obtain the best-fit power exponent. (**Below**) PSD of the time series created by SKM, disabling the interaction K=0. Even in this case, 1/f fluctuations are observed without synchronization. This fact is further analyzed in the next section. In this calculation, we considered a set of sine waves with adjacent frequencies in the range 1 Hz around the base frequency 100 Hz.

We simulate the resonating acoustic signal $\phi_{a}\left(t\right)$ and its PSD, experimentally adopting the 500 eigenfrequencies from the lowest, as well as M=20. As before, the PSD of the original signal $\phi_{a}\left(t\right)$ reflects the eigenfrequencies and is always flat in low-frequency regions. On the other hand, the PSD of the absolute values of the signal $\phi_{a}\left(t\right)$ shows 1/f fluctuations as in Figure 5 above. This demonstrates that the resonance of the eigenfrequencies exhibits 1/f fluctuations. However, of course, we cannot generally claim that the resonance of a hall creates 1/f fluctuations or the supervene construction of the Großer Musikvereinssaal (Goldener Saal). In reality, concert hall resonance depends on complex factors such as the actual hall geometry, absorption by seating, diffuse reflections, and wall materials, so we cannot claim that the analysis here is universally applicable. Generalizing the generation of 1/f spectra by concert halls remains a topic for future research.

This resonance can also be expressed by SKM. Inputting many accumulating frequencies $\omega_{i}$ and disabling the interaction K=0, we obtain the frequencies agitated by the random resettings. In Figure 5 below, we demonstrated PSD for the accumulating frequencies. As always, the raw waveform retains the flat spectral shape, while the absolute values of the data clearly exhibit a 1/f-like spectrum over a wide frequency range.

### 4.4. Interpretation

This supports the hypothesis that amplitude modulation from acoustic resonance plays a significant role in shaping the temporal structure of solo performances. As with synchronization-based AM, the 1/f structure only becomes evident after demodulation, reinforcing the central role of nonlinear envelope extraction in revealing 1/f fluctuations.

Such modulation mechanisms are ubiquitous in the acoustics found in caves, temples, cathedrals, and natural environments, contributing to the universal presence of 1/f fluctuations in sound. Some examples are shown in the Appendix A.

## 5. Integration of Synchrony and Resonance: Not a Dichotomy

Thus far, we have presented synchronization and resonance as two distinct physical mechanisms for generating amplitude modulation (AM), which, in turn, leads to 1/f fluctuations upon demodulation. In this section, we emphasize that these mechanisms are not mutually exclusive; rather, they often coexist and interact synergistically in natural and musical systems.

### 5.1. Unified View of Frequency Accumulation

Both synchronization and resonance serve as processes of frequency accumulation. Synchronization aggregates oscillators around a mean frequency through dynamic coupling. Resonance enhances energy transfer at particular frequencies through structural filtering.

Each mechanism shapes the amplitude envelope of a waveform, leading to low-frequency AM components. These components manifest as 1/f fluctuation only after demodulation. Even in orchestral unison, performers play in acoustic spaces that resonate with their sound. Likewise, a solo singer naturally synchronizes breathing and phrasing with accompanists or room acoustics.

### 5.2. SKM Description of Frequency Accumulation

The spread spectrum of eigenfrequencies of the hall, instruments, and human body can be expressed by the set of frequencies $\omega_{i}$, and the natural nonlinearity of the system necessarily introduces the finite interaction *K* in SKM Equation (8).

Further complications arise from the tone color, or timbre, which is intrinsically associated with the instruments or the singers [3]. The sound color is determined by the unique combination of harmonics or overtones present in a sound wave, along with their relative intensities and how they change over time. Moreover, time delay and nonlinear frequency changes at the wall reflection, or the spatial extension of the instruments in the orchestra, may affect resonance and synchronization.

Contrary to the above uncontrollable settings, musicians actively control the frequency vibrations (vibrato), chest-voice/head-voice/humming resonance, and glissando/legato, making the resonance and synchronization processes quite complicated.

We cannot perform a systematic analysis including the above effects in this paper. Instead, we aim to demonstrate how resonance and synchronization are incorporated into SKM. As we have already discussed, KM itself cannot describe the intermittent on-and-off synchronization and cannot yield 1/f fluctuations.

### 5.3. Phase-Amplitude Interaction Map

We examined the extent to which both resonance and synchronization cooperatively yield 1/f fluctuations. The strength of synchronization is expressed in the coupling strength *K* in SKM and that of resonance in the number of oscillators N.

**Figure 6:**
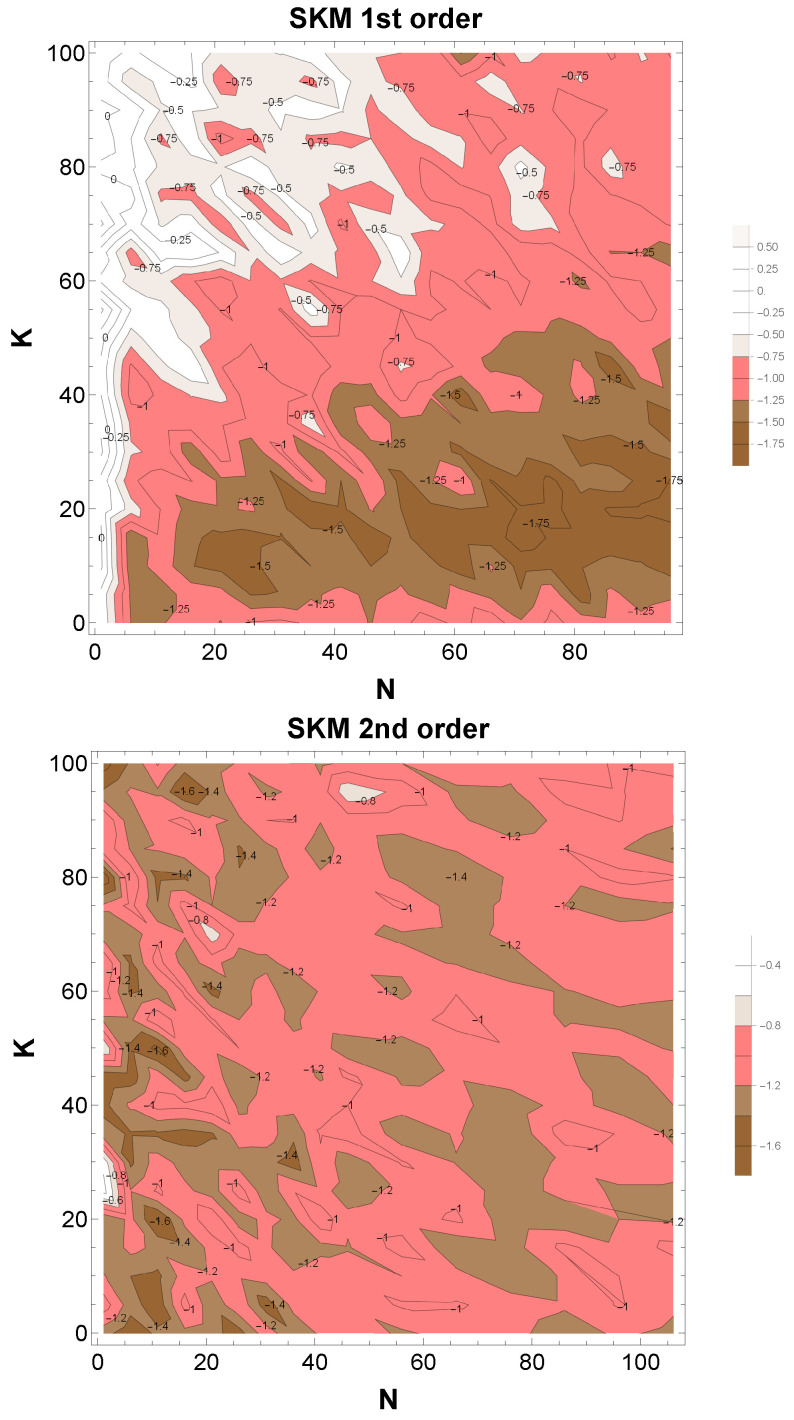
Density plots of PSD indices calculated from stochastic Kuramoto models (SKM) of the first order (**above**) and the second order (**below**) by changing the parameters N:1∼100 and K:0∼100. The 1/f fluctuations regions, marked by pink (indies ∼−1±0.2), are widely observed among Brown noise regions (brown) and white noise regions (white). (**above**) In this first-order SKM case, the 1/f fluctuation is manifest around the diagonal (K∼N). These cases correspond to the cooperation of synchronization and resonance. Interestingly, 1/f fluctuations are also observed in another region, beyond the valley from those lines, along with the horizontal axes (K∼0). This case corresponds to the resonance-dominant region for 1/f fluctuations. (**below**) The case of second-order SKM. The region of 1/f fluctuation is extended further than the first-order SKM.

In Figure 6 above, we plot the power PSD indices of the time series created by the first-order SKM Equation (8), varying the parameters *K* and *N*. As is evident, 1/f fluctuations, with indices around −1, naturally arise for N≈K, i.e., when resonance and synchronization balance. On the other hand, stronger resonance N>K yields smaller power indices, resembling Brownian motion, while stronger synchronization N<K yields larger power indices, resembling white noise.

In Figure 6 below, we plot the same for the second-order SKM Equation (9). Comparing the PSD-indices distributions of the two figures, it appears that the region of 1/f represented by the second-order derivative is wider, but since the number of derivatives in the equations is different, a simple comparison would not be possible.

The above results show that the 1/f fluctuation region is wide in both SKM models. The order parameter Equation (7) wildly fluctuates all the time Figure 4. Therefore, the distinction between synchronized and unsynchronized regions is meaningless. Thus, we propose the concept of “dynamical synchronization”, characterized by continual cycles of synchronization and resynchronization. (This is also exactly the case of economics, where many economic indices exhibit 1/f fluctuations. Likewise, in this economic case, an enormous amount of money and goods circulate endlessly with interaction, repeating cycles of synchronization and crash.) This perspective reframes 1/f fluctuation not as the result of a single dominant mechanism but as an emergent signature of systems where multiple forms of convergence cooperate.

### 5.4. Conclusions

The dichotomy between synchronization and resonance is artificial and misleading. Many real-world systems exhibit features of both. Our framework acknowledges their interplay and offers a more comprehensive model for understanding the origin of 1/f fluctuations.

## 6. More on Music

We decomposed music into three layers in the Introduction: (A) Structure (music score), (B) Physiology (performance), and (C) Physics (sound pressure). In this paper, we have mainly focused on layers (B) and (C), setting aside layer (A). We now explore layer (A) and another aspect of layer (B) from a much wider perspective. We will trace back to the origins of music, proceeding sequentially from our primary analysis (C), first discussing (B), and then (A).

### 6.1. More on Layer (B)

In layer (B), all players synchronize both in timing and in unison. The latter has already been studied above using the Kuramoto model. The former, temporal synchronization, can also be studied by extending the Kuramoto model. The physics is the same as in human posture control. In this case, we can use the equation derived from the following Lagrangian:

(15) $$L=\frac{1}{2}\sum_{i=1}^{N}{\dot{s_{i}}}^{2}-\frac{\mu }{2}\sum_{i=1}^{N}{\left(B_{3}.s_{i}\right)}^{2}-\frac{\lambda }{2N}{\left(\sum_{i=1}^{nspin}\left(B_{1}.s_{i}\right)\right)}^{2},$$

where each ‘spin’

(16) $$s_{i}=\left(\sin\left(\theta_{i}\left(t\right)\right)\cos\left(\phi_{i}\left(t\right)\right),\sin\left(\theta_{i}\left(t\right)\right)\sin\left(\phi_{i}\left(t\right)\right),\cos\left(\theta_{i}\left(t\right)\right)\right)$$

represents the direction of each part of the human body, and $\theta_{i},\phi_{i}$ are the latitude and longitude from the standing direction. Representation $B_{3}=\left(0,0,1\right),B_{1}=\left(1,0,0\right)$ is based on the same coordinate system. Applying the variational method to this Lagrangian, we obtain a set of differential equations for each ‘spin‘. The first term in Equation (15) represents the kinetic term, the second term tends to push all the spins up, and the third term tends to minimize the inclination toward the x-direction.

The set of equations derived from Equation (15) naturally exhibits 1/f fluctuations in a wide parameter space, provided the data is squared. Apparently, the basic mechanism is amplitude modulation due to synchronization. We want to extend this model in relation to the timing in layer (B). For example, by replacing $B_{3}=\left(0,0,1\right)$ by $B_{3}=\left(0,0,\sin\left(\omega t\right)\right)$, we may simulate the whole player group to follow a conductor. We will report these analyses in our future study and establish the AM/DM model for the origin of 1/f fluctuations in layer B.

### 6.2. More on Layer (A)

The musical score is a creative output of the human brain. Composers learn musical form from the vast body of music from the past, honing their skill to express unique artistic structures. Through this long training, composers coherently connect successful past attempts to complete their own artistic works. This process parallels human creative development: learning from past patterns and integrating them coherently.

Let us write a mathematical formula that expresses this dynamics, using an analogy with the Variational Autoencoder generative model [21], in which one learns a latent structure from many (*N*) past data points and coherently generates new data from those distributions. We propose the equation

(17) $$\frac{d^{2}\theta \left(t\right)}{dt^{2}}=-\omega^{2}\theta \left(t\right)+\frac{K}{N}\sum_{j=1}^{N}\sin\left(\theta \left(t-\tau_{j}\right)-\theta \left(t\right)\right),$$

where θ(t) represents some potential parameter that develops new data. This model can be called the Self-Synchronized Kuramoto model (SSKM). Simply put, human learning and creative development are self-organizing processes that synchronize with past experiences (especially successful ones) and consistently keep generating new behaviors based on their probabilistic structure.

This Equation (17) is not merely a whim, but is consistent with the findings of recent computational neuroscience research. In cognitive and creative activities such as musical composition, neural dynamics are shaped not only by instantaneous sensory inputs but also by repeated internal access to past experiences. Recent computational neuroscience work formalizes this idea by showing that hippocampal replay can train cortical generative models (including variational autoencoder-type architectures) that reconstruct and recombine sensory episodes, thereby supporting memory construction and imagination [22]. Human neuroimaging further indicates that replay-like internal sequences contribute to ongoing inference in hippocampal–prefrontal circuits [23]. Further, recurrent network models of planning emphasize a two-way interaction in which hippocampal replays are triggered by—and in turn adapt to—prefrontal dynamics [24]. In parallel, research on memory search models retrieval as foraging in a structured state space, characterized by locally coherent exploration punctuated by strategically timed switches (i.e., intermittent “jumps”) between clusters of related memories [25,26]. More broadly, the constructive memory literature stresses that episodic recall can flexibly recombine elements of past experiences to simulate novel events, providing a cognitive substrate for creative recombination [27].

Motivated by these converging perspectives, we introduce Equation (17) as a minimal dynamical representation of self-referential synchronization relevant to both brain function and compositional behavior. In this formulation, the current phase variable is coupled to multiple delayed replicas of its own past states, representing the internal reactivation of memory traces across different timescales. Stochastic resetting and phase diffusion account for exploratory transitions analogous to switching between memory clusters during search [25,26]. While Equation (17) is not intended as a detailed neural circuit model, it captures a generic computational principle shared by replay-based learning and memory-foraging accounts: Coherent output can be generated through repeated alignment with one’s own history, intermittently disrupted by exploratory departures. This mechanism naturally produces robust 1/f-type fluctuations, providing a bridge between cognitive/compositional dynamics and the synchronization-based origin identified in musical performance and other complex systems.

Equation (17) naturally exhibits 1/f fluctuations in a wide parameter space, provided the data is squared. Apparently, the basic mechanism is amplitude modulation due to synchronization. Probably, the rectification mechanism is intrinsic in the learning process. We want to study these dynamics in relation to layer (A) in our future publications and to establish the universal origin of 1/f fluctuations in all layers of music A–C.

## 7. Conclusions and Outlook

In this work, we proposed a mechanism for the emergence of 1/f fluctuations in music rooted in two fundamental physical processes: amplitude modulation (AM) and demodulation (DM). Through theory, simulation, and cross-domain analysis, we demonstrated that (a) AM arises from two main sources: synchronization among oscillators (e.g., orchestral unison) and resonance with environmental structures (e.g., solo performance in acoustic spaces). (b) DM, implemented through nonlinear transformations such as squaring, is essential for revealing latent 1/f spectral properties. (c) The AM/DM mechanism explains a wide range of 1/f phenomena in music, nature, and astrophysics. (d) It is often claimed that the order parameters make it possible to capture the essence of the system dynamics by disregarding fluctuations in individual components. However, in reality, order parameters exhibit large fluctuations that behave like 1/f in the low-frequency range [28].

### 7.1. Outlook

**Figure 7:**
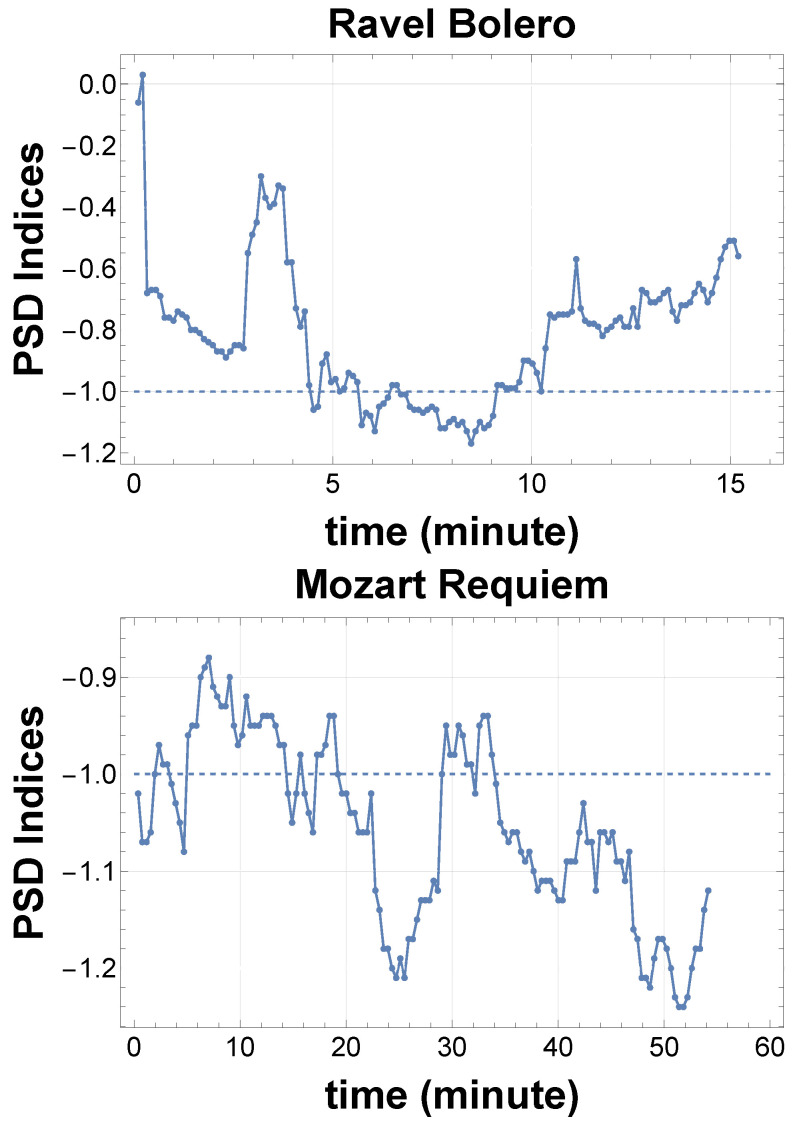
Examples of the time series of PSD indices around −1. These analyses are useful for extracting the synchronization history of the system. (**above**) PSD indices time series for music, Ravel’s Bolero [32], squared and analyzed in 2 min segments, sliding every 6 s. The history of PSD indices represents the degree of synchronization and does not reflect the sound volume. (**below**) PSD index time series for music, Mozart’s Requiem [33], squared and analyzed in 7 min segments, sliding every 21 s.

The AM/DM framework opens several directions for future research:

1. Acoustic effects: We considered some simple features of music and sound. In reality, music is full of delicate sound effects that may affect the low-frequency fluctuations through resonance and synchronization. These include the tone color or timbre, time delay, spatial extension of sound field, vibrato, humming, glissando, legato, etc. Furthermore, the recorded sound may be processed using various techniques, including reverb, chorus, delay, and distortion. We should integrate all of these to achieve complete resolution of the musical 1/f fluctuation.
2. Music pink noise from frequency modulation: We emphasized amplitude modulation (AM) in this paper and concentrated on the music performance. However, frequency modulation (FM) and other types of modulation may also yield a long-period structure from the individual short-period fluctuations. We want to explore 1/f fluctuations in music from a much wider perspective [8,9,10,29].
3. PSD time series: As the sound data is rich in data points, we can obtain local PSD indices by cutting the whole data into segments. These time series of PSD indices are useful for analyzing variations in the system’s synchronization and resonance. As shown in Figure 7, we can detect a clear transition in musical mood.
4. Spatial 1/f fluctuation: We can extend the ordinary notion of 1/f fluctuation in the time domain to the spatial domain as well: $k^{-3}$ fluctuations for the wave number *k*. A natural approach is to use the Complex Ginzburg–Landau Equation (CGLE) [30], the original equation of the Kuramoto model before phase reduction. In this case, as in the time domain, spatial resonance and synchronization may characterize the long-distance correlations and $k^{-3}$ fluctuations.

We must add another example of the frequency accumulation that we have not discussed in this paper. The primordial cosmic density fluctuations exhibit this type of spatial 1/f fluctuations, known as the Harrison–Zeldovich spectrum. They are derived from the infrared divergence [4,31] in the same way as the electric current in QED [7].

### 7.2. Final Thoughts

Often treated as a mysterious or random phenomenon, 1/f noise emerges here as a natural consequence of structured temporal processes. Our model emphasizes that apparent complexity often arises from the interaction of simple, interpretable mechanisms: oscillation, modulation, and nonlinear transformation. Music offers an intuitive manifestation of this principle, yet it represents only one expression of a general dynamic.

We hope this framework inspires further interdisciplinary exploration, where art and physics converge through the lens of temporal structure.

## Figures and Tables

**Table 1:** PSD exponents for the exponentiated data.

odd n-th power	1	3	5	7
slopes	0.84	−0.20	−0.15	0.09
even n-th power	2	4	6	8
slopes	−1.27	−1.15	−0.89	−0.68

**Table 2:** PSD exponents for nonlinearly transformed data. PSD of the first 20 s of sound data from Tchaikovsky’s Symphonie no 6 en si mineur, “pathétique” for Strings [11]. Here, 1/f fluctuation is exhibited after all possible demodulation procedures, while it is not exhibited in the original sound data. H() is the Heaviside function. μ is the mean of |#|, and # is individual original data value.

Transformation	PSD Indices
Original Signal: #	0.25
Squared Signal: ${\#}^{2}$	−1.25
Absolute value: |#|	−1.3
Rectification: Max(#,0)	−1.28
Negative-rectification: Min(#,0)	−1.27
thresholding above μ: # H(#−μ)	−1.24
anti-thresholding below mean but positive:	
# Max(# H(μ−#),0)	−0.89
thresholding timing: H(#−μ)	−1.1

**Figure A1:**
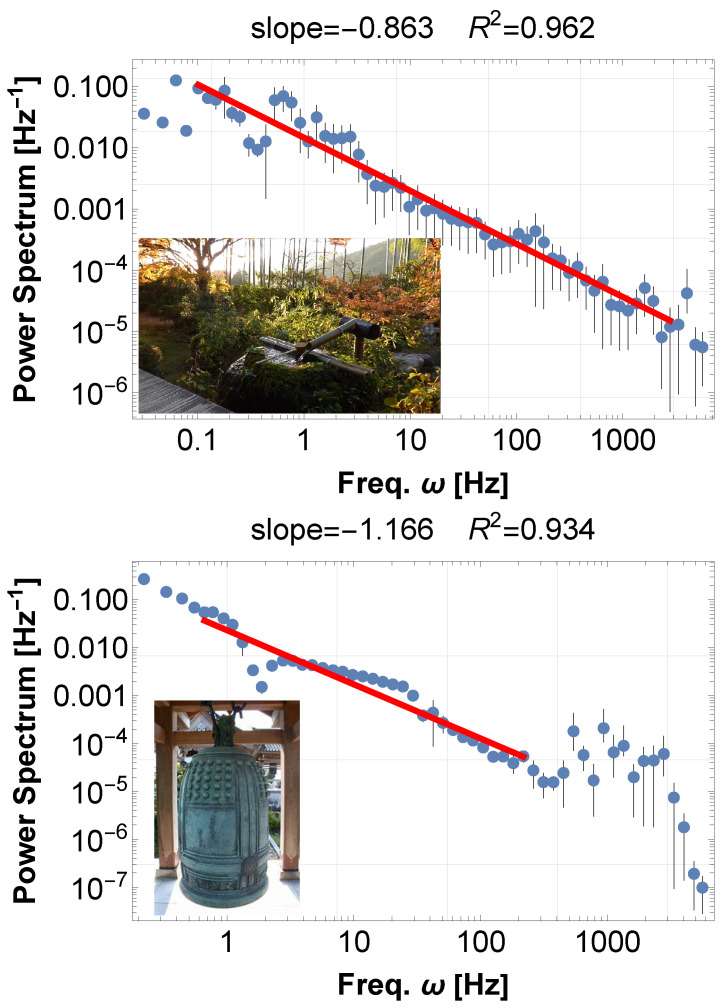
Kyoto sounds in temples. Sound resonance of devices in temples yields 1/f fluctuations. The data is squared before PSD analysis. (**above**) PSD of the sound data recorded on the Suikinkutsu, the water-harp cave at Hosen-In, Kyoto. The water from the basin is designed to drip into a 2 m Mino-Yaki ceramic pot buried in the ground. The dripping water resonates and reverberates in the pot’s hollow, producing a clear melody like a koto. Perpetual water drips activate the eigenfrequencies of the pot, like the random resetting triggers individual oscillators $\theta_{i}$ in the Kuramoto model. (**below**) PSD of the Big Bell sound data recorded at Enko-Ji temple, Kyoto. An initial strong strike activates the Big Bell eigenmodes, which resonate and produce sound beats. This sound power emission resembles the energy emission by earthquakes; both are modulated by the wave beat of the system’s eigenoscillations.

**Figure A2:**
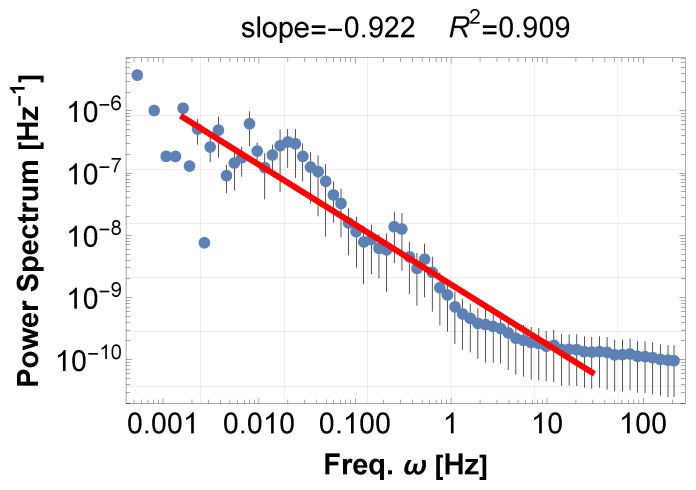
Cretan Sea sounds reflect the synchronization of many wave modes at the seashore. PSD of the Cretan Sea sound data recorded at the Orthodox Academy of Crete on 13 July, around 4 a.m. before the down. PSD shows a power index of −0.92, indicating an almost 1/f fluctuation, in addition to some wave-specific quasi-periodicities.

**Figure A3:**
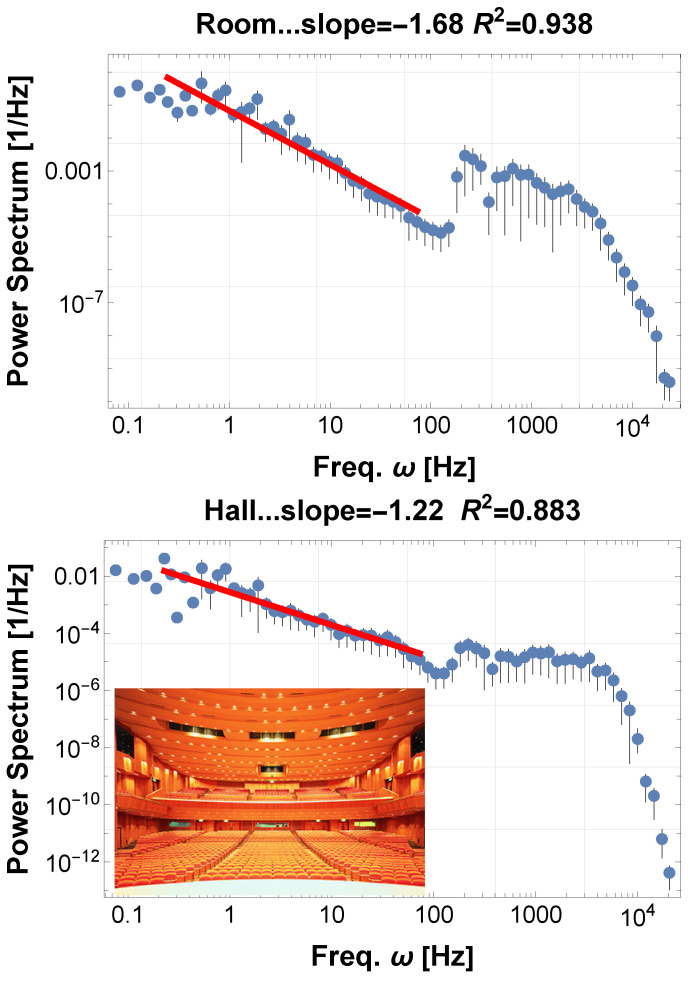
We first recorded the singing data, ’The Tulips Have Come into Bloom,’ in an ordinary room. (**above**) PSD obtained by replaying the recorded data in an office. The power index is −1.68, not 1/f fluctuations. (**below**) PSD obtained by replaying the recorded data on the stage in the Music Hall of Kyoto-Sangyo University. The PSD power index has changed to −1.22, approaching the 1/f fluctuations. We performed the same experiments, changing songs and styles, and found that PSD indices tend to approach −1 at this hall, although the indices were well below −1 in the office. The resonance of the hall may generally help to yield 1/f fluctuations.

## Data Availability

The raw data supporting the conclusions of this article will be made available by the authors on request.

## Appendix A. Case Studies in Acoustic and Natural Environments

In this appendix, we briefly introduce a series of case studies that highlight the versatility and explanatory power of the AM/DM framework. These real-world examples illustrate how 1/f fluctuations emerge in diverse acoustic settings and physical systems, extending beyond controlled laboratory or musical environments.

### Appendix A.1. The Water Harp Cave at Hosen-in, Kyoto

There is a variety of sound sources that yield 1/f fluctuations. The examples here are 1/f fluctuation originating from layer C of the music layer.

The Hosen-in temple in Kyoto features a “Water Harp Cave” that produces resonant sounds through underground water drips [34]. These perpetual water drops, falling into a hard ceramic vessel (Mino-yaki, approximately 2 m in diameter) buried in the ground, excite the cavity’s resonant modes. The result is an amplitude-modulated acoustic signal that, upon demodulation (e.g., via energy envelope extraction), reveals a 1/f spectral signature Figure A1 above.

Recordings from this site, when analyzed using squaring and spectral methods, show long-range correlation in the amplitude envelope, supporting our thesis that resonance and demodulation underlie 1/f fluctuation even in naturally occurring acoustic spaces.

Another soundscape is the Big Bell at the Enkoji campus [35] in the quiet northern Kyoto area. PSD shows 1/f fluctuations(Figure A1 below).

### Appendix A.2. Cretan Sea Soundscape

The recordings from the Cretan shore in Greece provide another compelling example. The periodic arrival and collapse of waves at the seashore at midnight yield a broadband acoustic signal.

Preliminary analysis indicates that after rectification or squaring, the amplitude fluctuations of this soundscape exhibit a 1/f spectrum on top of some periodic wave signals, Figure A2. This suggests that the marine acoustic wave naturally provides synchronization of many resonating waves that are favorable for the emergence of AM/DM-based 1/f fluctuation.

Although temporally and physically distant from musical systems, these signals obey the same structural principles outlined in our AM/DM model.

### Appendix A.3. Sound in Room and Hall

We recorded a singing voice in a room and replayed it in both an ordinary office and a professional music hall at Kyoto-Sangyo University. The PSD power index −1.68 in the office (Figure A3 above), changed to −1.22 in the hall (Figure A3 below). This change may directly reflect differences in resonance effects between a room and a hall.
